# Expanding Pharmacists’ Prescribing Authority and Medication Uptake: Evidence From Pre-Exposure Prophylaxis

**DOI:** 10.1016/j.focus.2025.100415

**Published:** 2025-08-13

**Authors:** Bita Fayaz-Farkhad

**Affiliations:** Annenberg School for Communication, University of Pennsylvania, Philadelphia, Pennsylvania

**Keywords:** Pharmacists’ prescribing authority, HIV preventive care

## Abstract

**Introduction:**

Considering the crisis in access to care in the U.S., pharmacies are expected to significantly expand access to a variety of preventive care, including HIV care. As of August 2024, 10 states allow pharmacists to initiate pre-exposure prophylaxis, a medicine taken to prevent HIV, independently. This study analyzed how the expansion of pharmacists’ authority to independently initiate pre-exposure prophylaxis affected pre-exposure prophylaxis prescription rates per 100,000 county residents.

**Methods:**

Hand-collected data on pharmacists’ prescribing authority from 2015 to 2023 were linked to county-level pre-exposure prophylaxis prescription rates. Using these data, the impact of the laws was estimated through a difference-in-differences approach. The analysis first calculated changes in pre-exposure prophylaxis prescription rates before and after the expansions and then compared these changes between counties in states that expanded pharmacists’ scope of practice (treatment group) and counties in states that did not (control group).

**Results:**

Expanding pharmacists’ prescribing authority increased pre-exposure prophylaxis prescription rates by 11.6%. The largest effects were observed in states that allowed pharmacists to prescribe pre-exposure prophylaxis without any mandatory training, a requirement that may otherwise burden pharmacists. Effects were most prominent in nonrural counties and counties with high insurance coverage and lower proportions of Black and Latinx populations.

**Conclusions:**

These results suggest that expanding pharmacists’ prescribing authority will not improve disparities in pre-exposure prophylaxis use if the laws fail to address structural barriers, such as staffing constraints or a lack of capacity.

## Highlights

• Expanding pharmacists’ pre-exposure prophylaxis prescribing authority raised prescription rates by 11.6%.
• Largest effects occurred in states with no mandatory pharmacist training.
• No gains were seen in counties with higher Black or Latinx populations.
• Results highlight gaps in pre-exposure prophylaxis access despite expanded pharmacy authority.

## INTRODUCTION

Despite impressive levels of protection in randomized placebo-controlled trials among those who receive pre-exposure prophylaxis (PrEP) for HIV consistently,^1^^,^^2^ the first decade of implementing PrEP has revealed a wide gap in adoption by demographic groups and across geographic regions.^3^^,^^4^ For example, in 2021, Black individuals accounted for 42% of new HIV diagnoses but only 14% of PrEP users in 2022. In contrast, White individuals made up 26% of new HIV diagnoses but 64% of PrEP users.^5^ Even more starkly, recent Centers for Disease Control and Prevention estimates have suggested that 94% of White individuals who could benefit from PrEP have been prescribed it, compared with just 13% of Black and 24% of Hispanic/Latinx individuals.^6^ Geographic disparities are also evident. For every new HIV diagnosis, 22 people are on PrEP in the Northeast, compared with only 10 in the South.^5^ These disparities are attributed to barriers such as limited knowledge, stigma, distrust of medical institutions, cost, and institutional factors.^7^, ^8^, ^9^, ^10^, ^11^, ^12^, ^13^, ^14^, ^15^, ^16^

In light of the crisis in access to HIV care in the U.S., the National HIV/AIDS Strategy emphasizes the role of pharmacies in increasing access to a variety of HIV prevention services, including PrEP.^17^ As of August 2024, 10 states allow pharmacists to deliver PrEP without a physician’s prescription.^18^^,^^19^ Although the accessibility of pharmacies and high trust in pharmacists are strong foundations for the potential success of expanding pharmacist prescribing authority,^20^ it is essential to quantify their efficacy empirically. This paper examines the causal relationship between expanded pharmacist prescribing authority and county-level PrEP prescription rates.

Pharmacist prescribing authority varies across states in 4 key ways.^19^ First, whereas many states allow pharmacists to prescribe PrEP for up to 60 days before requiring a referral to a primary care provider, others permit pharmacists to provide PrEP without time limitations.^19^ Second, states differ in whether they prohibit prior authorization requirements—a time-consuming process in which insurers require approval before pharmacists can dispense medication.^19^ Third, varying training requirements across states may pose burdens that limit pharmacists’ ability to take on expanded roles. Time constraints are a major obstacle to pharmacists assuming greater patient care responsibilities.^19^^,^^21^ Finally, whereas some states’ legislation includes guidance on services eligible for pharmacist reimbursement, others lack clear provisions for compensating pharmacists for PrEP services.

Although policymakers and healthcare leaders increasingly call for expanding the role of providers that offer HIV prevention services, concerns remain that pharmacy-based interventions may be less effective among lower SES populations, particularly those with reduced access to care or no health insurance, and among racial and ethnic minorities who often face greater structural barriers to care.^22^ Although emerging evidence shows that pharmacies are often more geographically accessible in these communities than traditional PrEP-prescribing clinics,^23^^,^^24^ many of these pharmacies may lack the resources needed to provide PrEP services. In particular, pharmacies in Black and Latinx neighborhoods may face limitations such as staffing shortages, inadequate training, limited inventory capacity, or financial barriers that prevent PrEP integration—despite the high community need.^25^^,^^26^ This suggests that allowing pharmacists to initiate PrEP may not improve disparities in PrEP use if the policy fails to address these structural barriers.

Past research on pharmacists’ prescribing authority suggests that expanding their prescribing role may increase PrEP use. One study found that expanding pharmacists’ authority to prescribe rescue inhalers to prevent and treat asthmatic symptoms and insulin pen needles to treat diabetes was successful.^27^ Even more relevant to the problem, a recent report compared the annual fill rate index for PrEP medications over time in states that expanded pharmacists’ prescribing authority with that in states that did not and found that such a change significantly increased PrEP use.^28^ This study builds on prior work by identifying which specific components of pharmacist prescribing authority (no quantity limit, no prior authorization, no mandatory training, and a legal requirement for reimbursement) facilitate the efficacy of pharmacist prescribing authority. This distinction is crucial given the wide variation in scope and implementation requirements across states. This study also extends prior analyses by examining heterogeneity in effects across racial and ethnic groups, different socioeconomic groups, and attitudes toward the lesbian, gay, bisexual, and transgender (LGBT) population using county-level demographic composition and state-level public opinion indexes as measures.

## METHODS

### Measures

This study gathered state-by-year data on pharmacists’ authority to initiate PrEP from the National Alliance of State & Territorial AIDS Directors.^19^ Appendix Table 1 (available online) lists the precise dates used in the analysis. Detailed information on pharmacist scope of practice regulations—including whether a state allows pharmacists to independently prescribe PrEP, limits on the quantity of PrEP a pharmacist may distribute, prohibitions on prior authorization, mandatory training requirements, and legal reimbursement provisions from 2015 to 2023—was collected. Five measures of pharmacist authority were Measure 1, any expansion in the pharmacists’ authority to prescribe PrEP independently; Measure 2, expansion without quantity limit; Measure 3, expansion without prior authorization requirement; Measure 4, expansion with no training requirement; and Measure 5, expansion with payment guideline.

To study the effects of prescribing authority for pharmacists on PrEP use, the number of individuals with at least 1 day of prescribed PrEP in a year per 100,000 county residents was obtained from AIDSVu for 2015–2023.^29^ One limitation of the data is that AIDSVu suppresses PrEP-use data for counties with fewer than 3 PrEP users or populations under 100 to protect personally identifiable information. When data were unavailable or censored, the number of PrEP users was assigned as zero, a choice that sensitivity analyses confirmed did not materially affect the results. A log transformation was applied to county-level PrEP prescription rates to account for skewness. These data were linked to the state-by-year data on pharmacists’ prescribing authority.

Subgroup analyses were conducted on the basis of 6 county- or state-level measures. These included (1) the proportion of the Black population, (2) the proportion of the Latinx population, (3) urbanicity level defined using the Rural-Urban Continuum Codes (RUCC) from the U.S. Department of Agriculture, (4) the uninsured rate, (5) designation as a Health Professional Shortage Area (HPSA) for primary care, and (6) state-level public opinion toward the LGBT community. Subgroup definitions were fixed using 2015 data to ensure that they remained time invariant throughout the study period. This approach avoids endogeneity that could arise if these characteristics changed over time in response to policy implementation. Data sources include the Area Health Resources Files,^30^ Health Resources and Services Administration, and EQUALDEX,^31^ a collaborative platform that compiles longitudinal public opinion estimates on LGBT and queer (LGBTQ) issues across counties and U.S. states.^31^ Detailed definitions and data sources for these variables are provided in Appendix Table 2 (available online).

Although the main policy of interest is pharmacists’ authority to prescribe PrEP, other PrEP and health policies were included in the models to ensure that concurrent policy changes do not confound the estimates. These include state Medicaid program benefits (e.g., removal of prior authorization regulations,^32^ coverage of telehealth-delivered PrEP, and coverage of targeted case management^33^). Furthermore, all models controlled for state Medicaid expansion, county-level data on per capita supply of physicians, hospitals, hospital beds, Federally Qualified Health Centers, median household income, unemployment rate, poverty rate, and managed care penetration rate, obtained from the Area Health Resources Files.^30^

### Statistical Analysis

This study uses a difference-in-differences (DID) design to compare changes in outcomes between treatment and control states before and after policy implementation. Treatment states are those that have implemented the policy, whereas control states have not implemented it during the study period. In particular, the analyses are based on the following model:

$$\log\left(Y_{ist}\right)=\beta_{0}+\beta_{DD}\left(1\left(t\geq t_{c}\right)\right)+\alpha_{s}\times 1\left(State_{s}\right)+\delta_{t}\times 1\left(Year_{t}\right)+[\delta_{s}\times 1\left(State_{s}\right)\times \left(t-t_{c}\right)\times 1\left(t<t_{c}\right)+\gamma_{x}\times X_{ist}+\epsilon_{ist}$$

where $Y_{ist}$ is the log transformation of county-level PrEP prescription rates in county i in state s and year t. $t_{c}$ is the implementation date of the policy of interest, and the binary variable $1(t\geq t_{c})$ indicates the postpolicy period. The coefficient of interest is $\beta_{DD}$. The model also includes a vector of time-varying state covariates, $X_{ist}$, and state- and year-fixed effects. The validity of the design was assessed by testing parallel pretreatment trends between the treatment and control groups.

As discussed in Data, 5 measures of policy change were created and used in separate regressions: (1) any expansion relative to that in comparison counties, (2) expansion without a prior authorization requirement relative to that in comparison counties, (3) expansion without quantity limit relative to comparison counties, (4) expansion without training requirements relative to that in comparison counties, and (5) expansion with payment guidelines relative to that in comparison counties.

To assess whether the effects of pharmacist scope of practice expansions varied across populations or settings, stratified analyses were conducted by county- or state-level characteristics. For each of the 6 measures described earlier, counties or states were classified into 2 groups. Specifically, separate DID models were estimated for the following:

1. Counties with above-median versus below-median Black population share;
2. Counties with above-median versus below-median Latinx population share;
3. Urban counties (counties with RUCC Codes 1–3) versus rural counties (counties with RUCC Codes 4–9);
4. Counties with high versus low uninsured rates;
5. Counties designated as partial or full HPSAs for primary care versus counties not designated as HPSAs; and
6. States with above- versus below-median public support for LGBT rights using public opinion scores from EQUALDEX, which reflect aggregated survey-based sentiment toward LGBTQ rights and individuals, scaled from 0 to 100, with higher values indicating more supportive attitudes.

Finally, the treatment effects were estimated by the year of the change to address concerns about staggered policy implementation.^34^, ^35^, ^36^ All analyses were conducted using Stata 18 (StataCorp LLC, College Station, TX).

## RESULTS

Table 1 contains the DID estimates of expanding pharmacists’ prescribing authority on county-level PrEP prescription rates. Panel 1 shows the estimated effects from a model that controls for year- and state-fixed effects, state- and county-level controls, and other state-level policies. Expanding pharmacists’ scope of practice increased PrEP prescription rates by 11.6%. Because county-level initiatives are relevant in the context of the analysis, Panel 2 includes county-by-year fixed effects to control for common shocks within a county. Estimates indicated a statistically significant increase of 10.2%, consistent with the results from Panel 1.

**Table 1:** Effect of Expanding Pharmacists’ Prescribing Authority

	Any expansion	No quantity limit	No prior authorization	No training requirement	With payment guideline
Panel 1					
Effect	0.116**	0.046	0.051	0.177**	0.045
	(0.05)	(0.06)	(0.06)	(0.07)	(0.05)
R-squared	0.842	0.839	0.837	0.836	0.84
*n*	26,533	24,045	23,590	24,065	23,974
Panel 2					
Effect	0.102*	0.037	0.007	0.150*	0.022
	(0.06)	(0.08)	(0.07)	(0.090)	(0.07)
R-squared	0.678	0.676	0.674	0.678	0.68
*n*	26,533	24,045	23,590	24,065	23,974
Panel 3					
Effect	0.090**	a0.061	0.061	0.156***	0.063**
	(0.03)	(0.04)	(0.04)	(0.04)	(0.03)
Lead	0.040	0.001	0.020	0.028	−0.002
	(0.05)	(0.06)	(0.06)	(0.04)	(0.05)
R-squared	0.854	0.85	0.85	0.848	0.852
*n*	23,710	21,495	21,086	21,506	21,427

*Note*: Level of significance: ***p<0.01, **p<0.05, and *p<0.1.

SEs in parentheses are clustered at the state level. All models include state- and year-fixed effects.

The identification strategy relies on the parallel trends assumption that PrEP prescription rates in counties without policy changes provide a valid counterfactual for those with expanded pharmacist authority. To investigate the validity of the pretreatment trend assumption, Panel 3 includes a 1-year lead of the treatment variable. The estimates for the lead of the treatment effect variable were not statistically significant, supporting the identification assumption.

**Figure 1:**
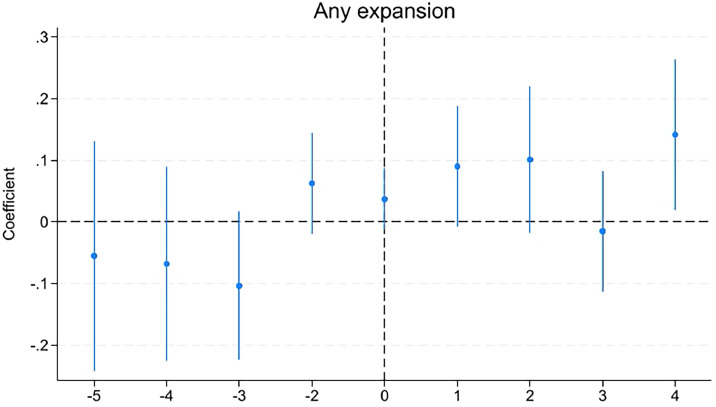
Effect of expanding pharmacists’ prescribing authority. *Note*: The figure displays the coefficients and their respective 95% CIs from a linear differences-in-differences model that includes state- and year-fixed effects. The vertical line represents the first year during the sample period when a state experienced an expansion in pharmacist prescription authority.

Figure 1 presents a graphical analysis of the effects of expanding pharmacists’ scope of practice in prescribing PrEP over time. Because every treated county has at least 1 year of data before the expansion in the sample, effects were estimated relative to the year before treatment, t= −1. Points left of the vertical line indicate the differences in treatment and control counties before the expansion in prescribing authority for pharmacists.

Prior to the expansion of practice authority for pharmacists, estimates were all statistically similar to zero, indicating that PrEP use trends in each group of counties did not differ before the change in laws. After implementation, PrEP prescription rates increased and remained consistently higher across the subsequent years, indicating a discrete and sustained policy effect rather than a gradual trend.

**Figure 2:**
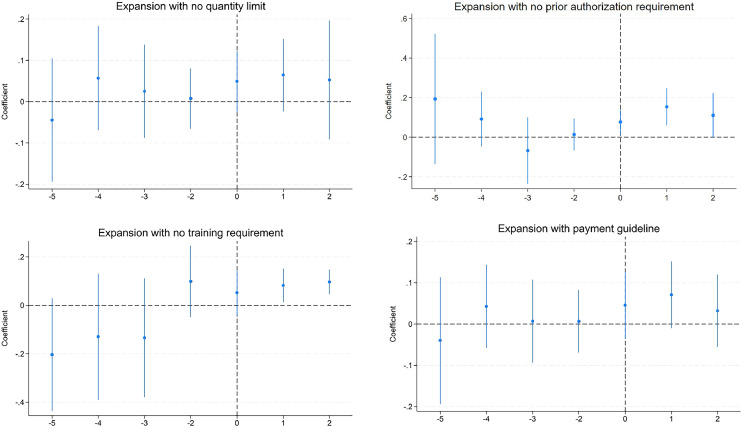
Effect of expanding pharmacists’ prescribing authority with different implementation conditions. *Note*: Each figure displays the coefficients and their respective 95% CIs from a linear differences-in-differences model that includes state- and year-fixed effects. The vertical line represents the first year during the sample period when a state experienced an expansion in pharmacist prescription authority.

Table 1 and Figure 2 evaluate whether different implementation conditions affect the outcome of interest. Although there was no detectable effect of expanding pharmacists’ prescribing authority without limitation on how much PrEP may be distributed to a single patient, removal of prior authorization requirements, or guidelines for reimbursements for pharmacists’ service, Column 4 shows that in states without training requirements, PrEP prescription rates increased by 17.7% after the scope of practice expansion.

A remaining question is whether the scope of practice laws had the same effects on PrEP prescription rates across different demographic groups or geographic regions. Appendix Table 3 (available online) and Appendix Figure 1 (available online) report the pattern in counties with rates above the mean versus below the mean rate of racial and ethnic minorities. The estimates in Appendix Table 3 (available online) indicate that PrEP prescription rates were more responsive to the policy change in counties with lower Black or Latinx population rates. Next, models were estimated across counties with higher rates of rural versus nonrural residents, counties with high versus low rates of insured population, and counties with a sufficient versus an insufficient number of healthcare providers. In Appendix Tables 4–6 (available online) and Appendix Figure 2 (available online), the estimates were larger for nonrural counties, counties with higher rates of insured population, and counties not designated as health professional shortage areas. Finally, models were estimated across states with high versus low public acceptance of LGBT, and interestingly, the effects were stronger in states with lower scores for LGBT friendliness (Appendix Table 7, available online). However, modeling the effect of expanding pharmacist prescribing authority in states with lower public acceptance of the LGBT community failed the pretrend test in Appendix Figure 3 (available online). As a result, estimates from this model should be interpreted with caution.

Appendix Table 8 (available online) presents estimates from 4 empirical approaches that consider different samples and functional forms to assess the sensitivity of the main results. Estimates were consistently positive and statistically significant at the 5% level in 3 of the 4 specifications and at the 10% level in all 4. The effect of allowing pharmacists to prescribe PrEP with no prior authorization requirement was statistically significant in only 1 of the 4 specifications. Similar to the main results in Table 1, there was no significant difference in the change in PrEP prescription rates in states that allowed pharmacists to prescribe PrEP independently with no quantity limit or in those states that enabled pharmacists to prescribe PrEP with payment guidelines.

In addition, the analysis assessed whether state- and county-level characteristics predicted the adoption of pharmacist PrEP authority to provide some reassurance that the scope of practice policies is not endogenous. Appendix Table 9 (available online) presents estimates for each economic and demographic control included in the main specification. All but 1 of the estimates were statistically insignificant.

Recent literature points out potential issues with DID analysis with the staggered implementation of a policy across states.^37^^,^^38^ It becomes challenging to identify a truly comparable control group for each treatment period. One way to address this concern is to decompose the treatment effects by the year of the change. Thus, a separate model was estimated for California, where the authority with legal payment requirements for pharmacists was expanded in 2019. Another model was estimated for Colorado, which, in 2020, expanded pharmacist authority to independently distribute unlimited quantities of PrEP and established legal requirements for pharmacist reimbursement. Likewise, models were estimated for Maine, Nevada, Oregon, Utah, and Virginia, which expanded their pharmacists’ prescribing authority in 2021 (in Maine and Nevada, insurers are required to cover PrEP prescribed by pharmacists; in Maine, insurers are prohibited from having prior authorization; in Nevada, pharmacists must complete a training program; in Utah, no quantity limitations apply). Finally, separate models were estimated for Arkansas, Illinois, and New Mexico, which expanded their pharmacists’ prescribing authority in 2023 (in Arkansas, Illinois, and New Mexico, pharmacists must complete a mandatory training program; in Illinois, insurers are required to cover pharmacist-initiated PrEP). These results are presented in Appendix Table 10 (available online). Consistent with earlier models, expansions without training requirements significantly improved PrEP prescription rates. When the effect of expansions with no prior authorization requirement was decomposed by the year of the autonomy change, only the treatment effects in 2021 were statistically significant. This result could be due to differences in statistical power across models.

Altogether, the results demonstrate that expanding pharmacist prescribing authority can improve PrEP uptake, particularly when policies are designed to reduce administrative burdens, although effectiveness remains limited in underserved and racially minoritized communities.

## DISCUSSION

This paper longitudinally modeled the impact of expanding pharmacists’ prescription authority for PrEP on PrEP prescription rates from January 2015 through December 2023. Consistent with the existing literature, the analysis confirmed that expanding pharmacists’ prescription authority for PrEP increased PrEP prescription rates, specifically in states that did not require mandatory training for pharmacists. These results are consistent with prior findings that show that healthcare providers, particularly those in resource-limited settings, are sensitive to regulatory and administrative burdens such as mandatory training.^21^^,^^39^^,^^40^ No significant change was observed in PrEP prescription rates in states that established legal requirements for proper pharmacist reimbursement, suggesting that pharmacists’ willingness to initiate PrEP may not be primarily driven by financial incentives. Rather, it may reflect a professional commitment to advancing public health, aligning with the ethical principle of beneficence. This aligns with qualitative findings from California, where pharmacists reported initiating PrEP out of a sense of community obligation despite a lack of reimbursement.^41^ No significant effect was observed for dispensing unlimited quantities of PrEP by pharmacists, suggesting that logistical barriers—such as patient demand, staffing limitations, or a lack of infrastructure—are more salient than regulatory limits in shaping prescribing behavior.

Although pharmacists can serve as another point of access to meeting the health needs of the general population, factors contribute to inadequate access to HIV preventive care among special populations, such as racial and ethnic minorities.^42^, ^43^, ^44^, ^45^, ^46^, ^47^, ^48^, ^49^ Notably, PrEP uptake did not increase in counties with higher proportions of Black or Latinx residents, indicating that expanded authority may not reach communities most impacted by HIV. This finding may reflect that pharmacies serving these communities are less likely to implement PrEP services—potentially owing to staffing constraints or a lack of capacity.^25^^,^^26^ Geographic disparities are also evident, with stronger effects in nonrural counties, counties with higher insured rates, and counties not designated as having a healthcare resource shortage. All in all, these results indicate that a more liberal scope of practice is likely to be less effective for individuals with lower SES, suggesting that people with lower income and education levels may experience fewer benefits from the policy owing to fewer resources in pharmacies that serve these communities and lower health insurance levels.

Although stigma around PrEP has been proposed as a barrier in communities with larger minoritized populations,^50^^,^^51^ the findings complicate that explanation. Indeed, the 3-way interaction analysis showed that the smaller policy effects occurred in counties with higher proportions of Black residents, but the strongest effects occurred in counties with both low public support for LGBTQ rights and smaller Black populations, suggesting that implementation challenges—such as pharmacy participation or resource constraints—rather than stigma alone are key drivers of impact. This result aligns with prior work suggesting that pharmacies, as neutral and less stigmatized environments, can enhance access to HIV prevention services because patients can access a range of services without being identified as seeking HIV care,^52^^,^^53^ but the findings suggest that without the infrastructure to support implementation, even affirming environments may not be enough to ensure access.

### Limitations

This study has several limitations. First, although the DID approach strengthens causal inference, unobserved state-specific trends correlated with both policy adoption and PrEP use may bias estimates. Second, the outcome data—sourced from AIDSVu—exclude counties with small PrEP user populations, potentially underrepresenting rural or low-access areas. Third, direct observation of whether pharmacists offered PrEP services after policy changes was not possible; instead, the estimates represent the potential opportunity to provide PrEP rather than the actual implementation. Finally, variation in implementation at the state level, such as differences between pharmacy chains and independents or local policy enforcement, could not be assessed and may have contributed to uptake heterogeneity.

## CONCLUSIONS

In summary, expanding pharmacists’ prescription authority increases the use of PrEP. However, the focus of policymakers is on reducing disparities. Although pharmacies and pharmacists offer many advantages for PrEP delivery to the most at-risk vulnerable population, this is not reflected in the subgroup analysis. Future research could address these challenges and create innovative policy tools to bridge these gaps.

## CRediT authorship contribution statement

**Bita Fayaz-Farkhad:** Conceptualization, Data curation, Funding acquisition, Writing – original draft, Writing – review & editing.

## Appendix Supplementary materials

Image, application 1

## Footnotes

Supplementary material associated with this article can be found in the online version at doi:10.1016/j.focus.2025.100415.
