# Supplementary material for: Expanding Pharmacists’ Prescribing Authority and Medication Uptake: Evidence From Pre-Exposure Prophylaxis
Source: AJPM Focus. 2025 Aug 13;4(6):100415. doi: 10.1016/j.focus.2025.100415 (PMC12547906; doi:10.1016/j.focus.2025.100415)
Supplement: Supplementary file 1 [file mmc1.docx]

**Appendix**

Figure A 1. Effect of Expanding Pharmacists’ Prescribing Authority, Across Racial and Ethnicity Groups

| Black   | Non-Black |
| --- | --- |
| Latinx   | Non-Latinx   |

*Notes*. Each figure displays the coefficients and their respective 95% confidence intervals from a linear differences-in-difference model that includes state and year fixed effects. The vertical line represents the first year during the sample period when a state experienced an expansion in pharmacist prescription authority.

Figure A 2. Effect of Expanding Pharmacists’ Prescribing Authority, Across SES Groups

| Non-Rural   | Rural   |
| --- | --- |
| Insured   | Uninsured   |
| Non-Shortage   | Shortage   |

*Notes*. Each figure displays the coefficients and their respective 95% confidence intervals from a linear differences-in-difference model that includes state and year fixed effects. The vertical line represents the first year during the sample period when a state experienced an expansion in pharmacist prescription authority.

Figure A 3. Effect of Expanding Pharmacists’ Prescribing Authority, Across Counties With More And Less Positive Attitude Toward LGBT People

| High Public Opinion Index   | Low Public Opinion Index   |
| --- | --- |

*Notes*. Each figure displays the coefficients and their respective 95% confidence intervals from a linear differences-in-difference model that includes state and year fixed effects. The vertical line represents the first year during the sample period when a state experienced an expansion in pharmacist prescription authority.

Table A 1. Timing of expansion in pharmacist prescribing authority

| State | Date |
| --- | --- |
| Arkansas | March 2023 |
| California | October 2019 |
| Colorado | July 2020 |
| Illinois | January 2023 |
| Maine | June 2021 |
| Nevada | October 2021 |
| New Mexico | April 2023 |
| Oregon | September 2021 |
| Utah | September 2021 |
| Virginia | December 2021 |

*Notes.* Data was collected by the authors from NASTAD.

Table A 2. Definitions of Subgroup Variables

| Measure | Definition | Source |
| --- | --- | --- |
| Black Population Rate | Percentage of county residents who identify as Black or African American. | Area Health Resources Files (AHRF) |
| Latinx Population Rate | Percentage of county residents who identify as Hispanic or Latinx. | Area Health Resources Files (AHRF) |
| Urbanicity Level | A 12-point scale classifying counties from highly urban (1) to highly rural (12), based on USDA Urban Influence Codes. | USDA Urban Influence Codes via AHRF |
| Uninsured Rate | Percentage of county residents without health insurance. | Area Health Resources Files (AHRF) |
| Underserved for Primary Care | Binary indicator for counties designated as Primary Care Health Professional Shortage Areas (HPSAs) by HRSA. | HRSA HPSA designations via AHRF |
| Public Support for LGBT Rights | State-level composite score measuring legal rights and public attitudes toward LGBT individuals (from EQUALDEX). | EQUALDEX Index (2022) |

*Notes*. Definitions and data sources for variables used in subgroup analysis. Variables were coded based on county or state-level characteristics from the AHRF, HRSA, and EQUALDEX datasets.

Table A 3. Effect of Expanding Pharmacists’ Prescribing Authority Across Counties With High And Low Racial and Ethnic Minorities

|  | Any expansion | No quantity limit | No prior authorization | No training requirement | With payment guideline |
| --- | --- | --- | --- | --- | --- |
|  |  |  | Black |  |  |
| Effect | 0.003 | -0.128 | -0.208** | 0.069 | -0.137 |
|  | (0.06) | (0.11) | (0.10) | (0.07) | (0.11) |
| R-squared | 0.88 | 0.88 | 0.879 | 0.876 | 0.881 |
| N | 13465 | 12029 | 11877 | 12241 | 11986 |
|  |  |  | Non-Black |  |  |
| Effect | 0.210*** | 0.166** | 0.203*** | 0.238*** | 0.168*** |
|  | (0.06) | (0.07) | (0.06) | (0.06) | (0.06) |
| R-squared | 0.73 | 0.7 | 0.709 | 0.715 | 0.708 |
| N | 13068 | 12016 | 11713 | 11824 | 11988 |

|  | Any expansion | No quantity limit | No prior authorization | No training requirement | With payment guideline |
| --- | --- | --- | --- | --- | --- |
|  |  |  | Latinx |  |  |
| Effect | 0.042 | -0.032 | -0.008 | 0.113 | -0.022 |
|  | (0.07) | (0.07) | (0.08) | (0.09) | (0.08) |
| R-squared | 0.882 | 0.879 | 0.879 | 0.878 | 0.881 |
| N | 13226 | 11870 | 11496 | 11771 | 11655 |
|  |  |  | Non-Latinx |  |  |
| Effect | 0.179*** | 0.064 | 0.113 | 0.257*** | 0.092 |
|  | (0.06) | (0.05) | (0.15) | (0.06) | (0.08) |
| R-squared | 0.739 | 0.745 | 0.743 | 0.742 | 0.745 |
| N | 13307 | 12175 | 12094 | 12294 | 12319 |

*Notes.* Standard errors in parentheses are clustered at the state level. All models include state and year fixed effects. Level of significance: ***<0.01, **<0.05, *<0.1.

Table A 4. Effect of Expanding Pharmacists’ Prescribing Authority Across Shortage and Non-Shortage Counties

|  | Any expansion | No quantity limit | No prior authorization | No training requirement | With payment guideline |
| --- | --- | --- | --- | --- | --- |
|  |  |  | Shortage |  |  |
| Effect | 0.057 | 0.027 | 0.07 | 0.165** | 0.039 |
|  | (0.06) | (0.06) | (0.06) | (0.06) | (0.06) |
| R-squared | 0.869 | 0.864 | 0.863 | 0.862 | 0.865 |
| N | 14197 | 13027 | 12320 | 12734 | 12930 |
|  |  |  | Non-Shortage |  |  |
| Effect | 0.171*** | 0.061 | 0.103 | 0.192** | 0.053 |
|  | (0.06) | (0.10) | (0.08) | (0.08) | (0.07) |
| R-squared | 0.752 | 0.752 | 0.758 | 0.757 | 0.759 |
| N | 12336 | 11018 | 11270 | 11331 | 11044 |

*Notes.* Standard errors in parentheses are clustered at the state level. All models include state and year fixed effects. Level of significance: ***<0.01, **<0.05, *<0.1.

Table A 5. Effect of Expanding Pharmacists’ Prescribing Authority Across Rural and Non-Rural Counties

|  | Any expansion | No quantity limit | No prior authorization | No training requirement | With payment guideline |
| --- | --- | --- | --- | --- | --- |
|  |  |  | Rural |  |  |
| Effect | 0.107 | -0.029 | 0.026 | 0.14 | -0.009 |
|  | (0.07) | (0.05) | (0.09) | (0.08) | (0.06) |
| R-squared | 0.628 | 0.632 | 0.632 | 0.633 | 0.634 |
| N | 13219 | 12017 | 12019 | 12313 | 12051 |
|  |  |  | Non-Rural |  |  |
| Effect | 0.069 | 0.045 | 0.038 | 0.189** | 0.044 |
|  | (0.07) | (0.06) | (0.07) | (0.08) | (0.06) |
| R-squared | 0.878 | 0.876 | 0.875 | 0.874 | 0.877 |
| N | 13314 | 12028 | 11571 | 11752 | 11923 |

*Notes.* Standard errors in parentheses are clustered at the state level. All models include state and year fixed effects. Level of significance: ***<0.01, **<0.05, *<0.1.

Table A 6. Effect of Expanding Pharmacists’ Prescribing Authority Across Counties With High And Low Rate of Uninsured

|  | Any expansion | No quantity limit | No prior authorization | No training requirement | With payment guideline |
| --- | --- | --- | --- | --- | --- |
|  |  |  | Uninsured |  |  |
| Effect | 0.12 | 0.024 | -0.037 | 0.145 | -0.062 |
|  | (0.07 | (0.10) | (0.09) | (0.09) | (0.07) |
| R-squared | 0.833 | 0.834 | 0.83 | 0.829 | 0.834 |
| N | 13160 | 12182 | 12122 | 12407 | 11903 |
|  |  |  | Insured |  |  |
| Effect | 0.115** | 0.064 | 0.105** | 0.197*** | 0.074 |
|  | (0.05) | (0.05) | (0.05) | (0.06) | (0.05) |
| R-squared | 0.857 | 0.851 | 0.852 | 0.85 | 0.853 |
| N | 13373 | 11863 | 11468 | 11658 | 12071 |

*Notes.* Standard errors in parentheses are clustered at the state level. All models include state and year fixed effects. Level of significance: ***<0.01, **<0.05, *<0.1.

Table A 7. Effect of Expanding Pharmacists’ Prescribing Authority Across Counties With More And Less Positive Attitude Toward LGBT People

|  | Any expansion | No quantity limit | No prior authorization | No training requirement | With payment guideline |
| --- | --- | --- | --- | --- | --- |
|  |  | Low Public Opinion Index | | |  |
| Effect | 0.253*** | 0.355*** | 0.155*** | 0.355*** | NA |
|  | (0.07) | (0.05) | (0.03) | (0.05) |  |
| R-squared | 0.782 | 0.783 | 0.78 | 0.783 | 0.781 |
| N | 13844 | 13182 | 13589 | 13182 | 12927 |
|  |  | High Public Opinion Index | | |  |
| Effect | 0.011 | -0.082* | -0.002 | 0.053 | -0.025 |
|  | (0.05) | (0.05) | (0.06) | (0.07) | (0.05) |
| R-squared | 0.872 | 0.87 | 0.871 | 0.866 | 0.87 |
| N | 12689 | 10863 | 10001 | 10883 | 11047 |
|  |  |  |  |  |  |

*Notes.* Standard errors in parentheses are clustered at the state level. All models include state and year fixed effects. Level of significance: ***<0.01, **<0.05, *<0.1.

Table A 8. Robustness Check, Effect of Expanding Pharmacists’ Prescribing Authority

|  | Any expansion | No quantity limit | No prior authorization | No training requirement | With payment guideline |
| --- | --- | --- | --- | --- | --- |
|  | Non-zero PrEP use counties | | | | |
| Effect | 0.120** | 0.056 | 0.054 | 0.180** | 0.053 |
|  | (0.05) | (0.06) | (0.06) | (0.07) | (0.05) |
|  |  |  |  |  |  |
| R-squared | 0.841 | 0.837 | 0.836 | 0.834 | 0.838 |
| N | 26109 | 23640 | 23199 | 23671 | 23577 |
|  | After dropping counties with | | | | |
| Effect | 0.123 | 0.008 | 0.018 | 0.190* | -0.016 |
|  | (0.07) | (0.08) | (0.06) | (0.11) | (0.06) |
|  |  |  |  |  |  |
| R-squared | 0.882 | 0.879 | 0.878 | 0.876 | 0.88 |
| N | 16517 | 15083 | 14675 | 15006 | 15035 |
|  | Poisson | | | | |
| Effect | -0.038 | 0.075 | 0.132*** | 0.118*** | 0.072 |
|  | (0.07) | (0.09) | (0.04) | (0.04) | (0.08) |
|  |  |  |  |  |  |
|  | |  |  |  |  |
| R-squared | 0.593 | 0.949 | 0.945 | 0.944 | 0.949 |
| N | 26533 | 24045 | 23590 | 24065 | 23974 |
|  | Negative Binomial | | | | |
| Effect | 0.059 | 0.074 | 0.070 | 0.132*** | 0.058 |
|  | (0.06) | (0.06) | (0.05) | (0.05) | (0.05) |
|  |  |  |  |  |  |
|  | |  |  |  |  |
| R-squared | 0.302 | 0.303 | 0.302 | 0.298 | 0.303 |
| N | 26533 | 24045 | 23590 | 24065 | 23974 |

*Notes.* Standard errors in parentheses are clustered at the state level. All models include state and year fixed effects. Level of significance: ***<0.01, **<0.05, *<0.1.

Table A 9. The Association Between State and County-Level Observables and the Likelihood of a State Expansion of Pharmacist Authority to Prescribe PrEP

|  | Coefficient | Std Err | Pvalue |
| --- | --- | --- | --- |
| Medicaid expansion | 0.107 | 0.109 | 0.331 |
| Whether state Medicaid prohibits prior authorization | 0.102 | 0.101 | 0.316 |
| Whether state Medicaid cover utilization management | -0.099 | 0.075 | 0.197 |
| Coverage of telehealth-delivered PrEP level 1 | 0.101 | 0.042 | 0.019 |
| Coverage of telehealth-delivered PrEP level 2 | -0.016 | 0.051 | 0.761 |
| Coverage of telehealth-delivered PrEP level 3 | -0.040 | 0.046 | 0.388 |
| Coverage of telehealth-delivered PrEP level 4 | 0.026 | 0.053 | 0.624 |
| Median household income | 0.001 | 0.001 | 0.320 |
| Poverty rate | -0.001 | 0.001 | 0.128 |
| Unemployment rate | 0.007 | 0.005 | 0.213 |
| Log(Hospital bed counts) | 0.001 | 0.001 | 0.162 |
| Log(Hospital counts) | 0.001 | 0.002 | 0.742 |
| Log(Physician counts) | -0.001 | 0.001 | 0.554 |
| Log(Federally qualified health center counts) | 0.001 | 0.002 | 0.78 |
| Medicaid managed care rate | -0.001 | 0.001 | 0.212 |

*Note.* Standard errors in parentheses are clustered at the state level. All models include state and year fixed effects.

Table A 10. Effect of Expanding Pharmacists’ Prescribing Authority By Timing of Policy Change

|  | Any expansion | No quantity limit | No prior authorization | No training requirement | With payment guideline |
| --- | --- | --- | --- | --- | --- |
|  | 2019 (CA) | | | | |
| Effect | 0.084 |  |  |  |  |
|  | (0.07) |  |  |  |  |
| R-squared | 0.846 |  |  |  |  |
| N | 22391 |  |  |  |  |
|  | 2020 (CO) | | | | |
| Effect | -0.008 |  |  |  |  |
|  | (0.05) |  |  |  |  |
| R-squared | 0.838 |  |  |  |  |
| N | 22469 |  |  |  |  |
|  | 2021 (ME, NV, OR, UT, VA) | | | | |
| Effect | 0.224*** | 0.178 | 0.155*** | 0.250*** | 0.1 |
|  | (0.05) | (0.14) | (0.05) | (0.05) | (0.08) |
| R-squared | 0.836 | 0.838 | 0.838 | 0.836 | 0.839 |
| N | 23633.000 | 22292 | 22365 | 23502 | 22496 |
|  | 2023 (AR, IL, NM) | | | | |
| Effect | -0.016 | 0.022 | -0.083 |  | 0.05 |
|  | (0.05) | (0.04) | (0.10) |  | (0.03) |
| R-squared | 0.837 | 0.838 | 0.836 |  | 0.839 |
| N | 23758.000 | 23096 | 22568 |  | 22821 |

*Note.* Standard errors in parentheses are clustered at the state level. All models include state and year fixed effects. Level of significance: ***<0.01, **<0.05, *<0.1.
